# Exploring noninvasive matrices for assessing long-term exposure to phthalates: a scoping review

**DOI:** 10.3389/fpubh.2024.1411588

**Published:** 2024-08-02

**Authors:** Li-wen Chen, Xin Chen, Hua-yan Mo, Chun-han Shan, Ruo-ping Zhu, Hui Gao, Fang-biao Tao

**Affiliations:** ^1^Department of Pediatrics, The First Affiliated Hospital of Anhui Medical University, Hefei, Anhui, China; ^2^Child Healthcare Department, Anhui Hospital Affiliated to Children’s Hospital of Fudan University/Anhui Provincial Children’s Hospital, Hefei, Anhui, China; ^3^Key Laboratory of Population Health Across Life Cycle (Anhui Medical University), Ministry of Education of the People’s Republic of China, Hefei, Anhui, China

**Keywords:** phthalate acid esters, nail, hair, noninvasive matrices, long-term exposure

## Abstract

The phthalic acid esters (PAEs) are one class of the most abundant and frequently studied pseudo-persistent organic pollutants. Noninvasive urine is an effective substrate for evaluating PAE exposure, but repeated sampling is needed to overcome this bias. This adds much work to on-site collection and the cost of detection increases exponentially. Therefore, the aim of this study was to conduct a scope review to describe the detection methods and validity of the use of other noninvasive matrices, such as nails and hair, for assessing long-term exposure to PAEs. The PubMed, Web of Science and China National Knowledge Infrastructure (CNKI), electronic databases were searched from 1 January 2000 to 3 April 2024, and 12 studies were included. Nine and three studies used hair and nails, respectively, as noninvasive matrices for detecting PAE exposure. Five articles compared the results of nail or hair and urine tests for validity of the assessment of PAE exposure. The preprocessing and detection methods for these noninvasive samples are also described. The results of this review suggest that, compared with nails, hair may be more suitable as a noninvasive alternative matrix for assessing long-term exposure to PAEs. However, sample handling procedures such as the extraction and purification of compounds from hair are not uniform in various studies; therefore, further exploration and optimization of this process, and additional research evidence to evaluate its effectiveness, are needed to provide a scientific basis for the promotion and application of hair detection methods for assessing long-term PAE exposure levels.

## 1 Introduction

Rapid agricultural and industrial development has resulted in significant exposure to potentially harmful chemicals, including pseudo-persistent organic pollutants such as phthalic acid esters (PAEs), bisphenols and organophosphates. These pseudo-persistent environmental pollutants have a relatively short biological half-life in the human body (1–4), but widespread environmental pollution and continued human exposure make them easy to detect in human biological samples. Take the PAEs, one class of the most abundant and frequently studied pseudo-persistent organic pollutant, as an example, people including pregnant women and children are generally exposed to these substances worldwide (5). It subsequently interferes with endocrine (6, 7) and immune functions (8, 9) and has significant effects on reproduction (10), neurodevelopment (1, 11), cardiovascular health (12) and even cancer development (9).

PAEs can be classified into low-molecular weight (LMW) phthalates and high-molecular weight (HMW) phthalates based on their molecular weight (13). LMW phthalates, such as dimethyl phthalate (DMP), dibutyl phthalate (DBP), and diethyl phthalate (DEP), are commonly used in nail polish, perfume, cosmetics, and pharmaceutical coatings. HMW phthalates, such as di(2-ethylhexyl) phthalate (DEHP), diisononyl phthalate (DiNP), di(noctyl) phthalate (DOP), and diisodecyl phthalate (DiDP), are mainly used in medical equipment, toys, buildings, food packaging and so on (13, 14). PAEs enter the circulatory system of the body mainly through oral ingestion, inhalation and skin absorption (15). According to the original study of the health effects of PAEs, blood is an ideal sample for assessing exposure to chemical pollutants. However, since it is an invasive sample, complications such as hematoma and pain may occur during collection (16), and it is difficult to collect in some special populations; thus, there are certain difficulties in practical application. Therefore, noninvasive surrogate matrices are necessary for assessing PAE exposure. In addition, PAE metabolites, whose half-life is usually approximately 12 h (1), are mainly excreted through the urine shortly after exposure. Therefore, urine is the best biological substrate for the assessment of exposure to these pseudo-persistent organic pollutants (17). There is a good correlation between urinary PAE metabolite concentrations and blood PAE metabolite concentrations (18). However, new problems arise. Although single random urine collection is convenient, it can lead to exposure misclassification bias, especially when large lifestyle and physical condition changes occur, such as pregnancy. Researchers have proposed collecting urine at multiple time points to overcome misclassification bias (19). There is evidence that even at just 9 months of gestation, multiple urine samples were collected to assess exposure, but the time variability was high (19). Moreover, the duration of repeated measurements is not uniform, and different measurement intervals may affect the study results and increase the amount of on-site sampling and related testing costs. Therefore, more economical, convenient and effective noninvasive matrices for assessing long-term PAE exposure need to be explored.

It has been suggested that nails are bioindicators that reflect long-term chemical exposure. In previous studies, nails were used more to detect metallic chemicals such as lead, mercury, zinc, copper, and iron (20). Several studies have shown that nails can be used to detect PAEs, and the concentration stability of PAEs in nails is much greater than that in urine within a certain period of time (21).

Hair is also a stable matrix. It starts in hair follicles, each of which has a capillary system at the root. During the growth phase of the hair shaft, chemicals in the serum bind to the hair and migrate into the hair (22). Thus, substances in the serum would theoretically be present in hair, making it a suitable matrix for assessing chemical exposure. Currently, hair is widely used to assess human exposure to metals, drugs, dioxins, polychlorinated biphenyls, and pesticides (23, 24), but research assessing PAE exposure is rare. In animal experiments, eight metabolites of DiNP were detected in the hair of rats chronically exposed to different doses of DiNP for 30 days. The levels of eight metabolites in hair showed a dose-dependent relationship with increasing exposure level. The results suggest that hair analysis is a better tool for assessing high-dose and long-term exposure (25). However, studies on the use of hair and nails as noninvasive matrices for assessing PAE exposure in the population are rare.

Therefore, the aim of this study was to systematically and comprehensively collect relevant published studies and conduct a scoping review to determine the detection methods and validity of long-term exposure to PAEs in nails and hair. The findings will provide clues as to whether noninvasive substrates such as hair and nails can be used to accurately assess long-term exposure to persistent organic pollutants.

## 2 Materials and methods

We conducted a scope-based review according to the five steps described by Arksey and O’Malley’s framework (26).

### 2.1 Defining the research question

Can hair and nails be used as noninvasive matrices for assessing long-term exposure to PAEs? If it is,

1. How do the results of PAE exposure assessed using nail/hair compare to the results assessed using blood/urine?
2. What sample handling procedures, such as extraction and purification, are used to detect long-term exposure to PAEs in hair and nails?

### 2.2 Search criteria

We conducted a systematic literature search for articles published from 1 January 2000 to 3 April 2024, using databases such as PubMed, the Web of Science, and the China National Knowledge Infrastructure (CNKI). The search terms used were subject headings and free words. The following terms were used in combination: “phthalates” (PAEs, “phthalate esters,” “phthalic acid esters”) and “detection” (test, monitor, determination, method, approach) “nail” “hair.” We also conducted a search of other relevant studies by reference.

### 2.3 Screening the target literature

According to the prespecified PECOS (Population, Exposure, Comparison, Outcome, Study Design) criteria (Supplementary Table S1), two authors (Li-wen Chen, and Xin Chen) identified eligible articles as follows: (1) published methodological studies for the detection of PAEs and their metabolites in human nails/hair, and (2) studies that simultaneously described the results of PAE exposure assessed by nail/hair vs. urine/blood; all observational studies (such as cross-sectional surveys, cohort studies, or case–control studies) were eligible.

Articles that met the following criteria were excluded: (1) if the data were from the same population or overlapped, only the article with the largest sample size was included; (2) the study was a review, meta-analysis, report, letter, comment, etc.; and (3) the subjects were animals, water, soil, etc., not humans.

### 2.4 Extraction of data

Hua-yan Mo and Chun-han Shan used a unified information extraction table for data extraction according to the purpose of the research. The following data were extracted: author, publication date, biological matrix, sample size, outcome indicators, etc. When there was disagreement, three or more authors discussed and voted according to the PECOS criteria, and opinions with a turnout of more than 50% were retained.

### 2.5 Summary of results

We report this review in accordance with the Preferred Reporting Items for Systematic Reviews and Meta-analyses (PRISMA) guidelines-Scope Review Extension (27). We present a narrative synthesis of the methods used to assess long-term exposure to PAEs using nails and hair and their validity.

## 3 Results

### 3.1 Included studies

**Figure 1:**
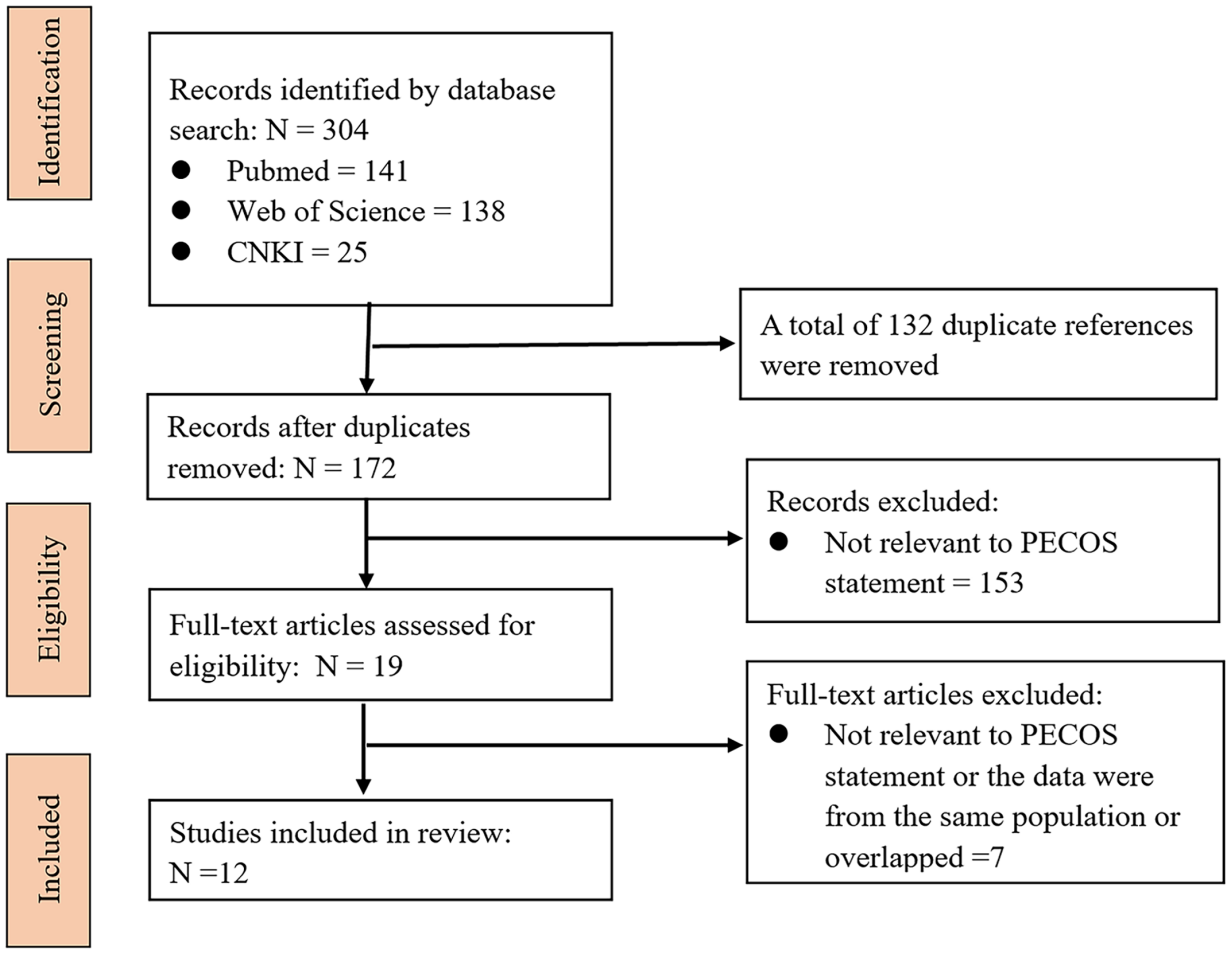
Flow chart of the literature screening process.

Through the systematic search strategy, 304 articles were initially identified. We excluded 132 duplicate articles based on the same unique identifier in PubMed. Then, according to the PECOS statement, 153 articles were excluded after browsing the titles and abstracts, and 7 additional articles were excluded after screening the full texts. Therefore, 12 eligible articles were ultimately included. The literature screening process is shown in Figure 1. For ease of reading, the PAEs and their metabolite name abbreviations that appear in this article are listed in Table 1.

**Table 1:** PAE and PAE metabolites and their abbreviations detected in nail, hair, and urine samples from 12 studies.

PAEs	Abbreviation	PAE metabolites	Abbreviation
Dimethyl phthalate	DMP	Monoethylhexyl phthalate	MEHP
Diethyl phthalate	DEP	Mono-(2-ethyl-5-hydroxyhexyl) phthalate	5-OH-MEHP
Diallyl phthalate	DAP	Mono-(2-ethyl-5-oxyhexyl) phthalate	5-oxo-MEHP
Diisopropyl phthalate	DiPrP	Monobutyl phthalate	MBP
Dipropyl phthalate	DPrP	Monoisobutyl phthalate	MiBP
Diisobutyl phthalate	DiBP	Monoethyl phthalate	MEP
Dibutyl phthalate	DBP	Monobenzyl phthalate	MBzP
Dipentyl phthalate	DPeP	Mono-(2-ethyl-5-carboxypentyl) phthalate	5cx-MEPP
Diisopentyl phthalate	DiPeP	Mono-(2-(carboxymethyl) hexyl) phthalate	2cx-MMHP
Butyl benzyl phthalate	BBzP	Mono-(2-propyl-6-hydroxyheptyl) phthalate	OH-MPHP
Dicyclohexyl phthalate	DCHP	Mono-(2-propyl-6-carboxyhexyl) phthalate	Cx-MPHxP
Diisohexyl phthalate	DiHP	Mono-(2-propyl-6-oxyheptyl) phthalate ester	oxo-MPHP
Dihexylphthalate	DHP	Monomethyl phthalate	MMP
Dibenzyl phthalate	DBzP	Monoisononyl phthalate	MiNP
Diheptyl phthalate	DHeP	Mono-(2-ethyl-5-carboxyhexyl) phthalate	MECPP
Diisodecyl phthalate	DiDP	Monoctyl ethyl phthalate	MnOP
Dimethoxyethyl phthalate	DMEP	Monoisononyl phthalate	MiNP
Di-4-methyl-2-pentyl phthalate	DMPP	Monoisopropyl phthalate	MiPrP
Di-2-ethoxyethyl phthalate	DEEP	Monocyclic hexyl phthalate	MCHP
Bis (2-butoxy) ethyl phthalate	BBEP	Monohexyl phthalate	MHxP
Diethylhexyl phthalate	DEHP	Mono-2-heptyl phthalate	MiHeP
Diphenyl phthalate	DPHP	Mono-n-butyl phthalate	MnBP
Dioctyl phthalate	DOP		
Dinonyl phthalate	DNP		

### 3.2 Overview of the characteristics of the included studies

Eight studies used hair as a noninvasive matrix for detecting PAE exposure, three studies used nails as a noninvasive matrix, and one study used both hair and nails as detection samples (28). Two articles have compared the results of nail and urine tests as noninvasive matrices for the assessment of PAE exposure (29, 30). Two articles compared the evaluation results of hair with those of urine (31, 32). One article reported PAE metabolite concentrations in hair, urine, and nails (28). No articles comparing the assessment results of nails/hair with blood were found. Information on the included studies is provided in Table 2.

**Table 2:** Basic characteristics of the articles included in this review.

References	Country	Sample size	Matrix	Analyte	Outcome
Alves et al. (33)	Belgium	10	Nail	MEHP, 5-OH-MEHP, 5-oxo-MEHP, MBP, MiBP, MEP, MBzP	US-DLLME method development, nail PAE metabolites concentration
Chang et al. (34)	China	10	Hair	MEHP, 5-OH-MEHP, 5-oxo-MEHP, 5cx-MEPP, 2cx-MMHP	Hair PAE metabolites concentration, development of a measurement method
Martin et al. (35)	Germany	4	Hair	DMP, DEP, DBP, DEHP	Hair PAEs concentration, development of a measurement method
Yin et al. (36)		9	Hair	OH-MPHP, Cx-MPHxP, oxo-MPHP	Hair PAE metabolites concentration, development of a measurement method
Hsu et al. (37)	China	30	Hair	MEHP, MMP, MEP, MBP, MiBP, MiNP, MEHHP, MEOHP, MECPP	Hair PAE metabolites concentration, development of a measurement method
Luo et al. (38)	China	10	Hair	MEHP, 5-OH-MEHP, 5-oxo-MEHP, MEP, MnOP, MBzP, MiNP, MiBP	Hair PAEs and their metabolites concentration, development of a measurement method
				DMP, DEP, DiBP, DBP, DMEP, DMPP, DEEP, DPP, DHP, BBzP, BBEP, DEHP, DPHP, DOP, DNP	
Zhou (39)	China	93	Hair	MMP, MiHeP, MBzP, MBP, MCHP, MEP, MEHP, MHXP, MiBP, MiPrP	Hair PAEs and their metabolites concentration, development of a measurement method
				BBzP, DAP, DiPP, DPP, DBzP, DiBP, DBP, DCHP, DEP, DiHP, DHP, DHeP, DiPrP, DPrP, DMP, DPHP, DiDP, DEHP, DOP	
Tian et al. (28)	China	60	Urine, hair, nail	MMP, MEP, MnBP, MiBP, MEHP, MEHHP, MEOHP	PAE metabolites concentration in urine, hair and nail
Alves et al. (29)	Belgium	20	Nail, urine	MEP, MiBP, MnBP, MBzP, MEHP, 5-oxo-MEHP, 5-OH-MEHP	PAE metabolites concentration in urine and nail
Giovanoulis et al. (30)	Norway	61	Nail, urine	MMP, MEP, MiBP, MnBP, MBzP, MEHP, 5-oxo-MEHP, 5-OH-MEHP, MPHP	PAE metabolites concentration in urine and nail
Fäys et al. (31)	Luxembourg	16	Hair, urine	MMP, MEP, MBzP, MEHP, 5-oxo-MEHP, 5-OH-MEHP, 5-cx-MEPP, 2-cx-MMHP, OH-MPHP, oxo-MPHP, MiNP, OH-MiNP, cx-MiNP	PAE metabolites concentration in urine and hair
Li et al. (32)	China	53	Hair, urine	MBP, MEHP, MBzP, MEP, MMP	PAE metabolites concentration in urine and hair

PAE, phthalate acid ester; US-DLLME, ultrasound assisted extraction combined with dispersive liquid–liquid microextraction.

### 3.3 Sample collection and storage

Nail collection did not emphasize which finger the nail was to be collected, and the preservation methods used were inconsistent. Although both Tian et al. (28) and Alves et al. (29) described nails that were wrapped in aluminum foil and then stored, Tian et al. (28) also emphasized storage conditions at room temperature and protected them from light. Two other studies involving nails did not describe how the nails were preserved (30, 33). The hair collection area was mostly the posterior apex/occipital part of the head (28, 34, 35, 37, 39), and hair was collected mainly near the scalp (28, 35, 37–39). Most researchers described hair samples as needing to be wrapped in aluminum foil. However, there were differences in the final storage conditions, some were recommended to be stored at room temperature and away from light (28, 36), some were recommended to be stored at 4°C (37), some were placed in a −20°C refrigerator (32, 39), and some were stored in a − 80°C refrigerator (31). Detailed information is provided in Table 3.

**Table 3:** Method for determination of phthalate esters in nails/hair.

References	Sample collection and storage	Sample preparation	Analytical instrument	Quality control	Limit of detection (LOD), Limit of quantification (LOQ)
Alves, et al. (33)		Cleaning: acetone	UPLC-MS/MS	Levels of target analytes were analyzed in procedural blanks, four of which were extracted daily and injected simultaneously with nail extract.	Method LOQ: 2–14 ng/g
		Optimal extraction conditions: 180 μL trichloroethylene (extraction solvent), 2 mL trifluoroacetic acid (methanol), extraction time was 2 h, vortex time was 3 min	Electrospray ionization		Instrument LOQ: 0.02–0.92 ng/g
Chang et al. (34)	Collect hair from the back vertex of the head.	Dichloromethane disinfection.	HPLC-MS/MS	The linear analysis of 5 metabolites of DEHP in hair was carried out in the concentration range of 1-100 pg./mg, and 5 calibration curves were obtained. Relative internal standard values were used to estimate the matrix effect, avoiding the influence of the background level of the analyte from the primary hair. Repeat 3 times.	LOD:0.2–1.0 pg./mg
		Incubation solution: methanol/TFA (8.5:1.5, v/v) at 45°C	Analysis column: LUNA C18 column, filled with 3 μm particles (50 mm × 2.0 mm)		LOQ:1.0–5.0 pg./mg
		Extraction solution: Ethyl acetate (PH = 3)	Electrospray ionization		
Martin et al. (35)	Hair samples were cut from the posterior apex region of the head and, as close as possible to the scalp. All hair samples stored in the aluminum foil, stored at room temperature.	Cleaning: Milli-Q water and isopropanol	GC-MS	Triplicate ethyl acetate standard solutions were analyzed multiple times at six different concentration levels to establish multilayer calibration curves.	LOQ:0.005–0.080 ng/mg
		Incubation solution: 1 mL methanol/TFA (8.5:1.5, v/v) or 1 mL 2 M NaOH solution at 38°C			
		Extraction solution: 4 mL hexane/ethyl acetate (1:1, v/v; PH = 3)	Analytical column: HP-5MS (30 m × 0.25 mm, 0.25 μm)		
Yin et al. (36)	Hair samples with aluminum foil, avoid light preservation at room temperature.	Cleaning: Ultrapure water and acetone	LC-MS/MS	Matrix effects were assessed using blank matrices supplemented with native standards and internal standards. The concentration of the analyte in the program blank was subtracted from the concentration of the analyte in the sample extracted after extraction and then compared with the concentration of the analyte in the solvent.	Instrument LOD:9–153 pg./mL
					Method LOD:0.1–4.8 ng/g
		SPE: Samples were processed with methanol and phosphate buffer saline	Analytical column: (2.1 × 100 mm, 2.6 μm)		Instrument LOQ:29–510 pg./mL
		Reconstituted: 100 μL acetonitrile/ultrapure water (1:1, v/v)	Electrospray ionization		Method LOQ:0.2–6.5 ng/g
Hsu et al. (37)	Hair samples were collected from the posterior apex of the individual’s head. A hair sample 2 cm from the scalp was cut from the hair, wrapped in aluminum foil and placed in a paper envelope. Hair specimens were stored in a dark environment of 4°C.	Cleaning: water and acetone	LC-MS/MS	Validation experiments were performed using spiky mixed hair samples to prepare matrix-matched calibration curves for the quantification of phthalate metabolites in hair.	LOD:0.22–3.21 ng/g
		Solvent: TFA/methanol (5:95, v/v) or TFA/water (15:85, v/v), acetonitrile			LOQ:0.72–10.7 ng/g
		Redissolved solution: 100 μL of 50% (v/v) acetonitrile with 0.1% TFA.	Analytical column: reversed-phase LC column (Inertsil Ph, 5 mm, 150 mm × 4.6 mm)		
Luo et al. (38)	Collected all hair samples close to the scalp, wrapped with aluminum foil, then put them into a compact bag and stored in a dry and cool place.	Cleaning: Milli-Q water	LC-MS/MS	All glass equipment were soaked in lye (pH > 11) for more than 4 h, washed with tap water and ultra-pure water and dried in the oven, then roasted in the Muffle furnace at 400°C for 4 h, and washed and dried with dichloromethane and n-hexane before use. 4 groups of experiments were set up, including blank, matrix, blank labeling and matrix labeling, and each group had 3 replicates.	PAE metabolites:
					LOD:0.044–14.45 ng/g
		Solvent: 1 mL ethylacetate; after evaporation of ethylacetate, 4 mL mixed solution of hexane: acetone: ethyl acetate: acetonitrile (1:1:1:1, V/V/V/V)	Analytical column: Kinetex EVO-C18 100 A (2.1 mm × 100 mm, 5 μm)		LOQ:0.146–48.16 ng/g
			Electrospray ionization		PAEs:
		Re-dissolved solution: 200 μL methylalcohol	GC-MS/MS		LOD:0.048–364.7 ng/g
			Analytical column: DB-5MS capillary column (30 m × 0.25 mm, 0.25 μm)		LOQ:0.159–1,215 ng/g
Zhou (39)	Collection: stainless steel scissors (pre-cleaned with acetone); close to the scalp, preferentially cut the hair in the occipital region. Then, wrapped it in aluminum foil.	Cleaning: 0.1% SDS, ultrapure water	HPLC-MS/MS	Add 2 process blanks for every 22 samples, observe the possible contamination in the experimental process, and subtract the blank when the sample is quantified. Every 10 samples into a solvent blank, a fixed concentration of daily calibration standard before and after the instrument was used every day, to ensure the stable operation of the instrument.	PAE metabolites:
			Analytical column: Kinete Biphenyl		LOD:0.041–24 ng/g
	Save in the process of transportation: room temperature, protected from light and moisture.	Extraction solvent: acetonitrile	100A Column (100 × 2.1 mm, 2.6 μm)		PAEs:
	Final storage conditions: in the refrigerator at −20°C.	Redissolve: methylalcohol	Electrospray ionization		LOD:0.09–0.81 ng/g
Tian et al. (28)	Hair: collection: posterior apical region of the head, as close as possible to the scalp; approximately 3-5 cm in length	Hair and nails: cleaning: acetone, twice;	Online-SPE-LC-MS/MS	Linear standard calibration using standard solution. Laboratory contamination was checked by using a matrix labeled sample and a solvent blank sample in each batch of 10 samples. Assessment of contamination during sample preparation (pure water without any analytes as a blank control)	LOD: hair: 0.002–0.058 ng/g;
					Nails: 0.002–0.072 ng/g;
					Urine: 0.014–0.863 ng/mL
	Nail: collection: 10 samples. Nail clippers were washed with ethyl acetate, hexane, and dichloromethane and wiped with isopropanol before use.	Digest protein: 5 mL 1 M NaOH (−40°C)			LOQ: hair: 0.007–0.159 ng/g;
	Storage of hair and nails: wrapped in aluminum foil, sealed in polyethylene bags, stored at room temperature and protected from light	Extraction solvent: n-hexane and methyl tert-butyl ether (v/v, 1:1)	Analytical column: Poroshell 120 EC-C18 (4.6 mm × 100 mm × 2.7 μm)		Nails: 0.006–0.172 ng/g;
	Urine: morning spot-urine. Stored in polyethylene bottles at −80°C in the refrigerator.	The final extract was reconstituted in 500 μL methanol.			Urine: 0.036–2.508 ng/mL
Alves et al. (29)	The glassware needed for sampling was cleaned and heated in an oven at 450°C (overnight). Wrapped nails in aluminum foil and stored. The polypropylene vial for urine collection was washed with 10% HNO_3_ solution and methanol.	Nails: cleaning: acetone;	UPLC-MS/MS	Each analytical batch includes a set of procedure blanks, and each of 10 sample injection solvent blanks and calibration standard. The final concentration of the target chemical is the sample value minus the concentration of the target chemical in the corresponding procedure blank.	
		Extraction solvent: 2 mL TFA:methanol, trichloroethylene	Electrospray ionization		
Giovanoulis et al. (30)	Nail: collection: composite samples (hands); collected in paper envelopes	Nails: cleaning: acetone;	LC-MS/MS	Internal standard use: in the process of extraction, adding the internal standard, calibration was used to analyze the possible loss or change in the process.	
	Urine: collection: three samples (afternoon of day 1 and morning and afternoon of day 2); placed in high-density polyethylene bottles that had been washed with methanol	Extraction solvent: trichloroethylene			
	Save: −20°C freezer		Electrospray ionization	Matrix effect and recovery: in the process of method development, the evaluation to the matrix effect, and the recovery rate test, to ensure accurate extraction and determination of target compounds from a complex matrix.	
Fäys et al. (31)	Hair: collection: once at the end of each month for 6 months. Stored in aluminum paper in the refrigerator at −80°C.	Hair: cleaning: 5% SDS aqueous solution, methanol; extraction solvent: acetonitrile/HCl (80:20, v/v)	LC-MS/MS	Use of internal standards: in the process of extraction, adding the internal standard, calibration was used to analyze the possible loss or change in the process.	
	Urine: collection: random 1-3 times a week for 6 months	Re-dissolved solution: 100 μL 0.1% formic acid in water/acetonitrile (80:20, v/v)		Quality control samples: Both solvent blank and matrix addition samples were included in each batch of sample analysis to check for laboratory contamination and to ensure good performance of the assay.	
Li et al. (32)	Hair: collection: distal hair (approximately 30 g); the scissors were washed with acetone in advance	Hair samples were pre-washed and dried.	HPLC-MS/MS	The blank sample and spiking sample were analyzed throughout the whole analysis process. The final concentration of the target chemical is the sample value minus the concentration of the target chemical in the corresponding procedure blank.	
	Urine: morning spot-urine. Stored in a pre-cleaned glass container (100 mL)	Extraction solvent: ethyl acetate, dried.	Chromatographic column:		
	Save: − 20°C freezer	Re-dissolved solution: 300 mL of Milli-Q water:acetonitrile (40:60; v/v)	Agilent Eclipse plus C18 column (3.5 mm, 2.1 mm × 100 mm)		

PAEs, phthalate acid esters; TFA, trifluoroacetic acid; SPE, solid–phase extraction; UPLC-MS/MS, ultra-performance liquid chromatography–tandem mass spectrometry; HPLC-MS/MS, high performance liquid chromatography–tandem mass spectrometry; GC-MS, gas chromatography–mass spectrometry.

### 3.4 Preparation of samples

Effective cleaning and extraction techniques are important analytical methods for the detection of PAEs in biological samples. Before the nails and hair were used for analysis, they were first washed to remove contaminants such as dust from the surface. In the included studies, acetone was the solvent used for nail cleaning (28–30, 33). Compared with nails, hair washing solvents were significantly more diverse, and there was no uniform method. In the included articles, there were three main hair sample washing solvents: inorganic solvents (35–38), organic solvents (28, 31, 35–37), and surfactants (31, 39). The inorganic solvent was mainly ultrapure water (including Milli-Q water), the most commonly used organic solvent was acetone, followed by isopropyl alcohol and methanol, and the surfactant was an aqueous SDS solution. Detailed information can be found in Table 3. After cleaning, the nail/hair sample was ground to a powder for further manipulation. Most of the included articles did not specify what solvent was used to promote hair and nail dissolution prior to extraction. Martin et al. (35) reported that hair samples were incubated overnight with 1 mL of NaOH solution or 1 mL of methanol/trifluoroacetic acid (8.5:1.5, v/v) at 38°C prior to extraction.

The present study revealed that the handling procedures for nails and hair mainly included solid–phase extraction (SPE) (36, 37) and liquid–liquid extraction (LLE). Among the considered methods, trifluoroacetic acid/methanol is the most common organic solvent, followed by methanol, trifluoroacetic acid/water, acetonitrile, etc. The extraction solvent was different in different studies; for example, Alves et al. (33) found that the best extraction solution was trichloroethylene after experiments. Chang et al. (34) used ethyl acetate for LLE. Martin et al. used a mixed solution of n-hexane/ethyl acetate (1:1,v/v) (30) to perform LLE on preliminarily treated samples (35). Luo et al. (38) reported that a mixture of n-hexane:acetone:ethyl acetate:acetonitrile (1:1:1:1, V/V/V/V) was the most suitable extraction solvent after experimental adjustment. However, Xu suggested that acetonitrile is the best extraction solvent after experiments (39). See Table 3 for further details.

### 3.5 Analytical instrument

The use of analytical instruments is also crucial for the detection of PAEs in nails/hair. We found that the separation of PAEs and their metabolites in nails or hair was mainly carried out by chromatography. Chromatography is a separation technique that is coupled with a detector, such as ultraviolet or mass spectrometry (MS). Of the studies using hair samples for detection, four used only liquid chromatography–tandem mass spectrometry (LC-MS) for analysis (34, 36, 37, 39). One study used only gas chromatography–mass spectrometry (GC-MS) for analysis (35). However, in the study by Luo et al. (38), GC-MS/MS was used to detect PAEs in hair, and LC-MS/MS was used to detect PAE metabolites. LC-MS/MS was used for studies in which nails were used as the matrix (33). The details can be found in Table 3.

### 3.6 Comparison of PAE metabolite concentrations between hair/nail and urine samples

In a small sample study conducted by Alves et al. (29) in Belgium, ∑(MnBP, MiBP) and MEP were the major PAE metabolites detected in both urine and nails. They also performed correlation analysis and found that there was a significant correlation between different metabolites in nails and urine. The levels of MEHP in the nails were strongly correlated with the levels of ∑ (MnBP, MiBP; r = 0.73, *p* < 0.01) and MBzP (r = 0.52, *p* < 0.05) in the urine. There was a moderate correlation between 5-OH-MEHP and ∑ (MnBP, MiBP; r = 0.62, *p* < 0.01) and between 5-OH-MEHP and MEP (r = 0.56, *p* < 0.05). However, no significant correlation was observed for the same metabolites measured in either matrix. A study in Norway revealed that MEP was one of the main metabolites detected in nails and urine, and MnBP and MiBP were also found at relatively high concentrations in urine and nails (30). Except for MEP (r = 0.56-0.68, *p* < 0.001), no correlation was found between PAE metabolite concentrations in the nail and urine. Tian et al. (28) reported that the most common PAE metabolite in nails is MMP, followed by MiBP and MnBP.

Fäys et al. (31) reported that the three most common metabolites in hair were MEP, MEHP and MMP; MBP, MMP and MEHP in Li et al.’s study (32); and MMP, MEHP and MEP in Tian et al.’s study (28). The major metabolites detected in the urine of the three studies all contained MEP. Fäys et al. (31) showed that the MEP concentration in hair was significantly correlated with the MEP concentration in urine, but no correlation was found for other PAE metabolites. Tian et al. (28) reported no or only weakly significant correlations between PAE metabolites in nail, hair, and urine samples. The correlation coefficients of the PAE metabolite concentrations measured in these five studies for the same substances in different matrices are shown in Table 4.

**Table 4:** Comparison and correlation coefficient^#^ of PAE metabolite concentrations between hair/nail and urine samples.

PAE metabolites	Alves et al. (29)			Giovanoulis et al. (30)			Fäys et al. (31)			Li et al. (32)			Tian et al. (28)				
	Median (range)			Geometric mean (25th; 95th)			Median (range)			Geometric mean (50th; 95th)			[Mean (SD)]				
	Nail (ng/g)	Urine (ng/mL)	Correlation coefficient (Pearson)	Nail (ng/g)	Urine (Spot 1-3, μg/gcrea)	Correlation coefficients (Spearman)	Hair (pg/mg)	Urine (ng/mL)	Correlation coefficients (Spearman)	Hair (pg/g)	Urine (ng/L)	Correlation coefficients (Spearman)	Nail (ng/g)	Hair (ng/g)	Urine (ng/mL)	Correlation coefficients (Spearman)	
																Nail-Urine	Hair-Urine
MMP				89.7 (45.7; 814.1)	0.02–0.05 (−; 2.3-5.3)		<LOD (−, 62.3)	7.5 (<LOD, 1659.5)		10,700 (9,820; 58,000)	569 (551; 1,800)		21.1 (24.0)	98.6 (103)	21.5 (14.1)	0.07	0.17
MEP	146.3 (14.9, 976.5)	2.99 (0.85, 9.99)	−0.16	104.8 (38.9; 1873.0)	23.0-31.3 (5.7-10.6; 189.3-309.9)	**0.56–0.68** ^**^	7.9 (<LOD, 18,120)	49.8 (1.4, 6679.4)	**0.62** ^**^	2,450 (1,990; 7,250)	2,140 (2,810; 15,800)		5.69 (7.84)	19.8 (42.1)	73.1 (147)	−0.09	**−0.28** ^*^
MBP										27,200 (28,600; 198,000)	1,180 (1,260; 5,190)						
MiBP				19.9 (16.9; 69.8)	12.8-17.0 (7.2-11.0; 47.4-50)								9.33 (6.41)	14.5 (53.9)	46.6 (39.1)	0.04	0.03
MnBP				89.3 (59.4; 3272.3)	9.0-15.5 (4.8-9.7; 38.8-93.1)	0.09-0.24							7.41 (4.61)	10.3 (11.5)	228 (297)	0.11	0.14
Σ(MnBP, MiBP)	136.1 (38.6, 813.6)	6.67 (2.23, 28.66)	0.16														
MBzP	5.3 (<LOQm, 55.3)	0.80 (0.14, 4.48)	0.43	2.6 (1.5; 41.5)	3.3-4.2 (1.8-2.1; 15.5-26.1)		0.4 (<LOD, 5.8)	2.9 (0.1, 76.7)		1,340 (1,270; 1,890)	126 (103; 1,060)						
MEHP	87.4 (54.6, 858.6)	0.19 (<LOQm, 0.64)	−0.05	129.3 (62.3; 682.5)	−(−; 11.5-17.9)		12.1 (<LOD, 692)	1.6 (<LOD, 355.9)		1,490 (1,320; 26,207)	199 (163; 1,280)		6.44 (6.95)	60.5 (76.3)	8.25 (4.79)	**−0.31** ^*^	0.24
5-oxo-MEHP	<LOQm (<LOQm, 2.4)	0.39 (0.13, 0.74)	−0.09	0.21 (<LOQm; 1.9)	4.5-5.4 (2.8-3; 19.1–38)		0.11 (<LOD, 6.74)	3.9 (<LOD, 419.1)									
5-OH-MEHP	<LOQm (<LOQm, 12.9)	0.46 (<LOQm, 1.14)	−0.10	2.6 (0.65; 854.6)	5.1-5.7 (2.8-3.2; 20.3-30.6)		0.1 (<LOD, 1.84)	4.8 (<LOD, 591.5)									
MEHHP													0.27 (0.25)	0.72 (1.17)	38.9 (23.7)	**−0.27** ^*^	0.05
MEOHP													0.25 (0.15)	0.81 (2.94)	17.7 (10.7)	−0.13	−0.09
5-cx-MEPP							0.06 (-, 8.2)	5.4 (0.03, 495.0)									
2-cx-MMHP							—	2.0 (<LOD, 118.9)									
MPHP				49.0 (<LOQm; 775.9)	—												
OH-MPHP							−(-, 0.72)	<LOD (<LOD, 1494.7)									
oxo-MPHP							0.004 (-, 0.86)										
MiNP							—	1.3 (<LOD, 222.4)									
OH-MiNP							−(-, 2.88)	0.7 (<LOD, 132.0)									
cx-MiNP							<LOD (-, 35.6)	1.4 (<LOD, 212.2)									

PAE, phthalate acid ester; SD, standard deviation; LOQ_m,_ method limit of quantification; -, not calculated; LOD, limit of detection. ^**#**^In this table, only the correlation coefficients of the same PAE metabolite concentration between hair/nail and urine samples are shown. ^*^p < 0.05; ^**^p < 0.01. A bold value means that its corresponding *p* value is < 0.05.

## 4 Discussion

Overall, nails and hair may be a noninvasive matrices for assessing long-term PAE exposure levels. However, the evidence is insufficient, especially regarding the use of nail assessment in PAE exposure studies. The main solvents used for hair cleaning were water, acetone, isopropanol, and 0.1% SDS. In addition, sample handling procedures such as the extraction and purification of compounds from hair were not the same among the studies.

At present, six phthalates, namely, DMP, DEP, DBP, DOP, DEHP, and BBP, have been identified as priority control pollutants by the United States Environmental Protection Agency (USEPA). These compounds or their primary and secondary metabolites were mostly examined in these studies. Although nails and hair have been used as biological matrices for detecting PAE exposure, urinalysis is still the most common method.

After Alves et al. developed a method to analyze long-term exposure to PAEs in nails, few studies have used nails to assess long-term exposure to PAEs, and the results were mainly from their team. Their findings indicated that major PAE metabolites in nails and urine were similar, and specific PAE metabolites in nails and urine were significant correlated, suggesting that nails can be used as a noninvasive alternative matrix to assess long-term PAE exposure in humans (21, 29). Nonetheless, it has been argued that the use of nails is not suitable for reflecting intra-PAE exposure in humans. The theoretical concentrations of nail DiBP and DnBP estimated from pharmacokinetic models were much lower than the actual concentrations (40). Research by Giovanoulis et al. showed that participants who frequently used hand care products had higher concentrations of MnBP and MEP in their nails. After adjusting for other confounding factors, the use of more than 5 care products per day was significantly positively associated with the concentration of MEP in the nails (30). Therefore, PAEs or their metabolites in hand care products may increase the concentration of PAE metabolites in nails through direct penetration into nails. Nails are more likely to reflect external exposure than internal exposure (40). In addition, the study by Giovanoulis et al. did not find a correlation between nails and urinary MnBP or MiBP (30). The sample size of this study (*N* = 61) is currently the largest among studies exploring the feasibility of using nails to assess long-term exposure to PAEs. Theoretically, PAE incorporation into the nail occurs primarily through diffusion through blood, with the blood supply depositing PAEs or their metabolites into the germinal matrix and nail bed on the lower side of the nail plate, resulting in incorporation during nail formation (41). However, there are no studies on the comparison and correlation between nail and blood PAE metabolite concentrations. This finding needs to be further explored to determine the value of nail assessment of long-term PAEs exposure.

The use of hair as a noninvasive alternative matrix substrate for assessing long-term PAE exposure in humans has been studied more than the use of nails, but hair is more commonly used to monitor PAE exposure in vulnerable populations, such as newborns (42). This may be because the metabolic pathways reflected in hair are different from those in urine. For example, the metabolite profiles of DPHP in urine and hair are not the same, suggesting that metabolites often measured in urine may not be directly suitable for detection in hair (43). Second, because the steps of cleaning and extraction are not reasonable, PAEs and their metabolites may not be detected. Current studies have shown that most PAE metabolite concentrations in hair have no significant or weak correlation with PAE metabolite concentrations in urine (28, 31). Contaminants from external sources, such as chemicals in food and care products, may have an impact on these processes and thus affect the concentration of PAE metabolites in hair (44). In addition, only the study by Fays et al. evaluated the correlation of hair PAE metabolite concentrations with mean urinary PAE metabolite concentrations over time (31). Therefore, some scholars believe that to use hair analysis as a biological monitoring method for human exposure, it is necessary to clarify the absorption pathway of PAEs and their metabolic processes in hair, determine the relationship between exposure dose and PAE content in hair, and determine the relationship between PAE metabolite concentrations in hair and concentrations in urine (35). However, further research is needed.

Several researchers have studied methods for the detection of PAEs and their metabolites using hair as a matrix. Due to the use of shampoo and other products and air exposure, to better assess internal exposure to PAEs, it is necessary to wash hair before testing to reduce the impact of exogenous exposure (45). However, there is no unified scheme for the cleaning solvent used. The solvents used for investigator cleaning in our included studies included water, acetone, isopropanol, and 0.1% SDS. Zhou et al. (39) reported that the use of 0.1% SDS and ultrapure water to wash hair can better reduce the matrix effect and achieve a higher internal standard recovery rate. This finding is similar to the results of Martin et al. (46), who reported that the concentration of organic matter in hair after washing with organic solvents was lower than that after washing with water and surfactants. Similarly, a study by Zheng et al. (47) revealed that hair shafts of hair that had been washed with warm Milli-Q water appeared smooth under scanning electron microscopy, indicating effective removal of external contamination. The use of organic solvents as washing solvents may require consideration: During the washing process, impurities may be removed from the hair surface, and compounds of interest may also be extracted from the hair matrix, which will have an impact on the final analysis results (48). Therefore, water and 0.1% SDS may be more suitable washing solvents. Various studies have also used different approaches for sample handling procedures such as the extraction and purification of compounds from hair. Although some scholars have also explored the optimal extraction conditions during this study (38, 39), there is still a lack of data comparing with the use of other matrices to assess PAE exposure. These questions need to be explored further.

This study has important implications for our recent development of biological sample detection methods for the long-term assessment of pseudo-persistent organic pollutant exposure. However, there are some shortcomings that need to be carefully considered. For instance, this article only takes PAEs of the nonpersistent organic pollutants as an example. Different organic compounds have different chemical structures and volatilities, so the sample handling and detection methods may be different. For example, compared with the HPLC-MS/MS method, the GC-MS method is more suitable for detecting more volatile chemicals, and in the pretreatment derivatization is needed. In addition, the metabolic process involved in the detection of chemicals in the hair and nails, needs to be fully understood. This is important for selecting the chemicals to be tested. For example, PAE parent chemicals are very common in air pollution, and the use of products such as nail polish and shampoo inevitably causes hair and nail pollution. By washing hair and nails with organic solvents before grinding and measuring PAE metabolites, contamination problems can be avoided to some extent. However, it is not clear whether the organic solvent cleaning process can lead to the loss of chemicals in hair or nails, and thus the level of exposure is underestimated. The team is using neonatal hair and nails to develop tests to better avoid exogenous contamination, and looks forward to adding new evidence to the findings of this study.

## 5 Conclusion

The results of this review suggest that hair may be a noninvasive matrix for assessing long-term exposure to PAEs compared with nails. However, due to the lack of correlation between the concentration of PAE metabolites in hair and the average concentration of PAE metabolites in urine samples collected continuously over a period of time, the use of hair to assess long-term PAE exposure still needs further validation. Water and 0.1% SDS may be more suitable as washing solvents for the treatment of hair. However, handling procedures such as the extraction and purification of compounds from hair are not uniform in various studies; therefore, further exploration and optimization of this process and additional research evidence to evaluate its effectiveness are needed to provide a scientific basis for the promotion and application of hair detection methods for assessing long-term exposure levels of pesudo-persistent organic pollutant PAE.

## Author contributions

L-wC: Writing – original draft, Methodology, Formal analysis, Data curation. XC: Writing – original draft, Formal analysis, Data curation. H-yM: Writing – original draft, Data curation. C-hS: Writing – original draft, Data curation. R-pZ: Writing – review & editing. HG: Writing – review & editing, Funding acquisition. F-bT: Writing – review & editing, Conceptualization.
